# Effects of the hydroalcoholic extract of *Rosa damascena* on learning and memory in male rats consuming a high-fat diet

**DOI:** 10.1080/13880209.2017.1362010

**Published:** 2017-08-23

**Authors:** Arezoo Rezvani-Kamran, Iraj Salehi, Siamak Shahidi, Mohammad Zarei, Shirin Moradkhani, Alireza Komaki

**Affiliations:** a Neurophysiology Research Center, Hamadan University of Medical Sciences, Hamadan, Iran;; b Department of Pharmacognosy and Pharmaceutical Biotechnology, School of Pharmacy, Hamadan University of Medical Sciences, Hamadan, Iran

**Keywords:** Oxidative stress, passive avoidance, Morris water maze, antioxidant, flavonoid, lipid profile

## Abstract

**Context:** High-fat diet (HFD) can cause deficits in learning and memory through oxidative stress and increase Alzheimer disease risk. *Rosa damascena* Mill. (Rosaceae) extract possesses potent antioxidant properties.

**Objective:** This study investigated the effects of the hydroalcoholic extracts of petals of *R. damascena* on learning and memory in male rats consuming an HFD.

**Materials and methods:** Forty male Wistar rats (200–250 g) were randomly assigned to four groups: control, *R. damascena* extract, HFD and HFD + extract. The extract (1 g/kg bw daily) was administered by oral gavage for 1 month. Animals were allowed free access to high-fat chow for 3 months. The Morris water maze and the passive avoidance learning tests were used to assess learning and memory.

**Results:** In the passive avoidance learning test, the step-through latencies in the retention test (STLr) of the extract (147.4 ± 23.3) and HFD (150.3 ± 25.2) groups were significantly lower than those of the control group (270.4 ± 10.5) (respectively, *p* < 0.001 and *p* < 0.01). STLr was significantly higher in the HFD + extract group (265.3 ± 10.6) than in the HFD group (150.3 ± 25.2) (*p* < 0.01). Time spent in the dark compartment (TDC) in the HFD + extract group (5.3 ± 2.6) was significantly lower than that in the HFD group (85.8 ± 19.1) (*p* < 0.05).

**Discussion and conclusion:** Our results indicate that, while HFD or *R. damascena* extract alone leads to memory deficits, *R. damascena* extract exerted a positive effect on HFD-induced memory deficits. We hypothesize that the observed effects of *R. damascena* extract are likely due to its strong antioxidant properties.

## Introduction

The diets consumed by people in both developed and underdeveloped countries at present are high in fat and refined sugar. It is clear that such diets can negatively affect neuronal and brain functions (Molteni et al. 2002; Swinburn et al. 2004) and can damage cognitive function and increase dementia in Alzheimer’s disease patients (Gustaw-Rothenberg 2009; Walker and Harrison 2015). Diets that are high in saturated fat increase the risk of obesity and insulin resistance (Yki-Järvinen 2015). Endocrine abnormalities, including insulin resistance and high glucose levels, can cause damage to the middle temporal lobe and lead to cognitive decline and memory deficits (Greenwood and Winocur 2005). Increased glucose levels negatively affect the brain by increasing the expression of inflammatory cytokines, which leads to oxidative stress and increased cortisol release (Esposito et al. 2002). High-fat diet (HFD) consumption increases corticosterone levels, which can inhibit neurogenesis (Lindqvist et al. 2006). HFD can also lead to cognitive dysfunction by activating the oxidative and inflammatory signalling in the brain (White et al. 2009). A diet high in saturated fat decreases the levels of brain-derived neurotrophic factor (BDNF), which can lead to cognitive disorders and defects in learning and memory (Page et al. 2014). BDNF is a neurotrophin that is abundant in the hippocampus and the cortex (Musumeci et al. 2015) and has well-known effects on synaptic plasticity and neuronal excitation (Novkovic et al. 2015). HFD increases lipid peroxidation and leads to increased levels of malondialdehyde (Park et al. 2010; Ganji et al. 2017a). This can cause oxidative stress and learning and memory deficits (Park et al. 2010).

The *Rosa* genus (Rosaceae) includes 200 species and over 18,000 cultivars (Naquvi et al. 2014). *Rosa damascena* Mill. is called the Damask rose because it was originally brought to Europe from Damascus (Jalali-Heravi et al. 2008; Shamspur et al. 2012), though some sources list Iran as one of its origins (Tabaei-Aghdaei et al. 2007). *R. damascena* is one of the most popular plants in Islamic medicine and is used as a traditional remedy in Iran (Zargari 1992) because of its sedative (Rakhshandah and Hosseini 2006), anti-inflammatory (Hajhashemi et al. 2010), antibacterial (Özkan et al. 2004), analgesic (Hajhashemi et al. 2010) and antioxidant (Achuthan et al. 2003) effects. Extracts of this plant are also used in beauty products and perfumes in multiple countries (Özkan et al. 2004; Verma et al. 2011).

We investigated the neuroprotective effects of a hydroalcoholic extract of *R. damascena* on learning and memory in adult rats fed an HFD using two different behavioural tests. We tested the hypothesis that *R. damascena* extract attenuates memory and learning deficits caused by HFD, as measured by the passive avoidance learning and Morris water maze tests.

## Materials and methods

### Animals

**Figure 1.**
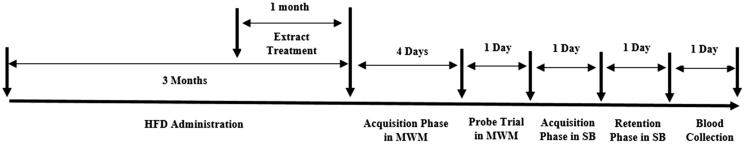
Timeline of experiments.

All animal procedures were conducted in accordance with guidelines established by the Research Ethics Committee at Hamadan University of Medical Sciences. Forty male Wistar rats (200–250 g) were purchased from Pasteur Institute (Tehran, Iran) and were housed in groups at 22 ± 2 °C under a 12 h light/dark cycle. The animals had free access to water and standard or high-fat rat chow. Animals were randomly assigned to four groups of 10 animals each: 1) Control (standard diet), 2) HFD, 3) HFD +1 g/kg bw hydroalcoholic extract of *R. damascena*, 4) Standard diet +1 g/kg bw hydroalcoholic extract of *R. damascena*. The extract (1 g/kg bw daily) was administered by oral gavage for 1 month (Trellakis et al. 2012; Joukar et al. 2013; Nazıroğlu et al. 2013). The experimental timeline is shown in Figure 1.

### Chemicals and reagents

Ketamine and xylazine were purchased from Webster Veterinary Supply (Sterling, MA). Ethanol, Na_2_CO_3_ and CH_3_COOK were purchased from Merck Co. (Darmstadt, Germany). AlCl3, sodium cholate, atorvastatin, cholesterol, the 3-hydroxy-3-methylglutaryl-coenzyme (HMG-CoA) reductase assay kit and Folin–Ciocalteu reagent were purchased from Sigma-Aldrich Co (St. Louis, MO). Hydrogenated oil and soybean oil were purchased from Sanati Behshar Co. (Behshar, Iran). Ghee was purchased from Damdaran Co. (Tehran, Iran). Sugar was purchased from Esfahansugar Co. (Esfahan, Iran).

### High-fat diet

Animals in the HFD and HFD + extract groups were fed an HFD composed of a standard diet (67.7%) with 8.3% ghee, 4.05% hydrogenated oil, 0.85% soybean oil, 0.8% sodium cholate, 1.0% cholesterol and 17.3% sugar (Matos et al. 2005). For concocting HFD, standard diet chows were powdered and this powder was mixed with a certain percentage of materials in the mixture and then came in the form of pellets. For feeding experiments, male rats were placed in cages and were fed with either standard diet (Behparvar, Iran) or HFD. Animals had free access to the HFD for 3 months. The compositions of the high-fat and standard diets are given in Table 1.

**Table 1.** The composition of the high-fat and standard diets.

High-fat diet (%)	Standard diet (%)
Standard diet 67.7%	Protein 21%
Ghee 8.3%	Fat 3.69%
Hydrogenated oil 4.05%	Carbohydrate 32.5%
Soybean oil 0.85%	Crud fibre 5.5%
Sodium cholate 0.8%	
Cholesterol 1.0%	
Sugar 17.3%	

### Preparation of extract

For the preparation of the hydroalcoholic *R. damascena* extract, *R. damascena* petals were purchased from a flower market (Hamadan, Iran) in spring 2015 and identified and authenticated by Dr. Ramazan Kalvandi at the Botanic Institute of the Hamadan University of Medical Sciences. A voucher specimen was deposited at the Department of Pharmacognosy and Biotechnology, School of Pharmacy, Hamadan University of Medical Sciences. These petals were dried in a room free of sunlight and then ground into a powder. This powder was then dissolved in 98% ethanol and extracted using distilled water and ethanol (1:1 v/v) as a solvent. This extract was filtered and concentrated under reduced pressure on a rotary evaporator. It was finally freeze-dried at −80 °C. The extract was dissolved in water and prepared fresh daily in our lab and 1 g/kg bw was administered daily by oral gavage for 1 month. Doses were chosen according to previously published data (Kheirabadi et al. 2008; Ramezani et al. 2008; Hajhashemi et al. 2010; Saxena et al. 2012).

The median lethal dose (LD_50_) of this plant extract has been reported previously (Lis-Balchin 2006; Esfandiary et al. 2015; Raghavendra et al. 2015; Mahboubi 2016). Oral LD_50_ of *R. damascena* and rose absolute was >5 g/kg in rats and dermal LD_50_ of *R. damascena* was >2.5 g/kg in rabbits (Lis-Balchin 2006; Mahboubi 2016). Consistent with results of another study, LD_50_ was determined 6 g/kg (Esfandiary et al. 2015). Additionally, it has been shown that an LD_50_ dose of 2 g/kg and higher than 2 g/kg is categorized as unclassified and therefore the extract is found to be safe (Raghavendra et al. 2015).

The potential toxic results of *R. damascena* infusion in dogs at doses 90–1440 mg/kg/day (0.5–8 times of human uses) for 10 successive days discovered a minimal nephrotoxic or hepatotoxic effect. Therefore, it might also have hepatotoxic consequences at extraordinary high doses (Akbari et al. 2013). In another experiment, the ethanol extract of *R. damascena* failed to show any mortality and toxic manifestations up to the dose of 3200 mg/kg (Raghavendra et al. 2015).

IC_50_ values of ethanol and watery extract of *R. damascena* have been discovered to be 18.46 and 22.1 μg/ml (Himesh et al. 2012). In another study, IC_50_ values of the extract in free radical scavenging was 2.24 μg/ml and in fat peroxidation assays was 520 μg/ml and fat peroxidation assays, respectively (Yassa et al. 2015).

### Determination of total phenolic

The total phenolic content of *R. damascena* was measured according to the method using the Folin–Ciocalteu reaction. Briefly, 1 mg of *R. damascena* was dissolved in 2.8 ml of deionized water, 2 ml of Na_2_CO_3_ (2%) and 0.1 ml of 50% Folin–Ciocalteu reagent. Afterward, the tube was incubated for 30 min at room temperature and the absorbance of the sample was measured at 750 nm against blank. Finally, total phenolic content was calculated equivalents of gallic acid per gram of extract.

### Determination of total flavonoid levels

Total flavonoid content was measured by colorimetric assay using aluminium chloride according to a formerly published method (Ordonez et al. 2006). Briefly, 1.0 mg/ml of extract was mixed with 2.8 ml of deionized water, 1.5 ml of 95% alcohol, 0.1 ml of 10% AlCl_3_ and 0.1 ml of 1 M CH_3_COOK. The mixture was then incubated at room temperature for 40 min and measured at 415 nm against a blank (deionized water). Flavonoid content was calculated as mg equivalents of quercetin per gram of extract.

## Behavioural studies

### Morris water maze test

The Morris water maze test was used to assess spatial memory (Asi et al. 2011; Asadbegi et al. 2017). A black circular pool (180 cm in diameter and 60 cm in height) was filled with water at 22 ± 1 °C to a depth of 25 cm. The pool was divided into four quadrants. A black escape platform was submerged 1 cm below the water level in the centre of the northern quadrant. The animals were placed in the water at one of the four randomly selected positions and the time between entry into the water and escape onto the platform (escape latency) was measured. Animals were trained with two blocks of four 60 s trials each at approximately the same time each day (10:00–12:00) for four consecutive days. The animals spent 30 s on the platform between each trial and were allowed to rest for 5 min between the two consecutive blocks. If an animal failed to locate the platform within 60 s, it was gently placed on the platform by the researcher and allowed to stay there for 30 s. A video camera (Nikon, Melville, NY) linked to a computer was mounted directly above the pool to record the escape latency, length of the swim path (distance travelled) and the time spent in the target quadrant. On the 5th day, each rat performed a single 60 s probe trial and a visible platform trial. No platform was present during the probe trials. In the visible platform trials, the platform was covered with aluminium foil. Escape latency, distance travelled and average swimming speed were recorded in each trial (Zarrinkalam et al. 2016).

### Passive avoidance learning (PAL) test

A standard passive avoidance conditioning apparatus was used for training and testing. The apparatus consisted of a shuttle box containing lighted and dark compartments. Each compartment measured 20 × 20 × 30 cm. A guillotine door that could be raised or lowered by the researcher separated the compartments. The floor of the apparatus consisted of stainless steel rods. A shock generator was connected to the floor rods of the dark compartment.

### Training

All animals were habituated to the experiment room for 1 h prior to testing. All training and testing procedures were performed between 10:00 and 14:00. Each animal was gently placed in the lighted compartment and allowed to habituate to the apparatus for 30 s, after which time the guillotine door was opened to allow access to the dark compartment. The rat has an innate preference for dark environments. After the rat entered the dark compartment, the door was closed, and the animal was allowed to remain for 30 s before removal to its home cage. The entrance latency to the dark compartment (step-through latency, STLa) was recorded based on the time the animal placed all four paws in the dark compartment. Animals that had not entered the dark compartment after 120 s were excluded from the experiment. The habituation trial was repeated after 30 min and was followed 30 min later by the first acquisition trial, in which the guillotine door was closed. During the acquisition trial, a 2 s foot shock (50 Hz, 0.8 mA) was delivered immediately after the rat entered the dark compartment. After 20 s, the rat was removed from the apparatus and placed in the home cage for 2 min. A sec acquisition trial was then performed as described above. Successful acquisition of the passive avoidance response was considered if the rat did not enter the dark compartment 120 s after being placed in the apparatus (Barzegar et al. 2015; Khodamoradi et al. 2015).

### Retention test

A retention test was performed 24 h after training to evaluate long-term memory. Each rat was placed in the lighted compartment, the guillotine door was raised after 5 s and the step-through latency in the retention test (STLr) and the time spent in the dark compartment (TDC) were recorded (Asadbegi et al. 2017; Ganji et al. 2017b). The test session ended when the animal either entered the dark compartment or remained in the lighted compartment for 300 s, indicating retention of the passive avoidance response. No electric shock was applied during the retention test (Khodamoradi et al. 2015).

### Plasma lipid profile measurements

At the end of the study, animals were anesthetized using ketamine/xylazine administered by intraperitoneal injection. After confirmation of anaesthesia by noting an absence of responsiveness to toe pinching, blood was collected by inferior vena cava puncture. Blood was centrifuged at 3000 rpm for 10 min at 4 °C. The plasma was obtained and sent to a clinical laboratory for measurements of lipid profile, such as triglycerides (TG), low-density lipoproteins (LDL), high-density lipoproteins (HDL) and cholesterol. The plasma concentration of TG, HDL and cholesterol were measured by colorimetric methods using the commercially available kits (Pars Azmun, Tehran, Iran), whereas LDL was calculated by Friedwald’s formula (Friedewald et al. 1972).

### HMG-CoA reductase activity

The HMG-CoA reductase inhibitory activity of the plants was determined based on spectrophotometric measurements. The concentration of the HMG-CoA reductase stock solution was 0.5–0.75 mg/ml. To measure of HMG-CoA reductase activity, 50 µg of the extract was used to determine enzyme activity using the HMG-CoA reductase assay kit (Sigma-Aldrich Co.). The reaction mixture was incubated at 37 °C, and absorbance was measured at 340 nm after 10 min. HMG-CoA reductase inhibitory activity was calculated using the following formula:

$$Inhibition\%\;=\frac{\Delta Absorbance\;control-\Delta Absorbance\;test}{\Delta Absorbance\;control}\;\times 100.$$

### Statistical analysis

Comparisons among the four groups were analyzed using analyses of variance (ANOVAs) followed by Tukey’s *post hoc* tests. The advantages of the Tukey method are that it tests all pairwise differences, it is simple to compute and reduces the probability of making a Type I error. Statistical analyses were performed using SPSS version 16.0 statistical software (SPSS, Chicago, IL). *p* < 0.05 was considered statistically significant. All of the results were expressed as mean ± SEM.

## Results

### Total phenolics, flavonoids, and enzyme activity

In this experiment, atorvastatin was used as a positive control, and distilled water was the negative control. Total phenolic and flavonoid contents of *R. damascena* were 3110.10 ± 10.20 and 1240 ± 14.5 mg per 100 g of extract, respectively. *R. damascena* had a 35% inhibitory effect on HMG-CoA reductase activity when compared with atorvastatin.

### Effects of *R. damascena* extract and HFD on lipid profile

**Figure 2.**
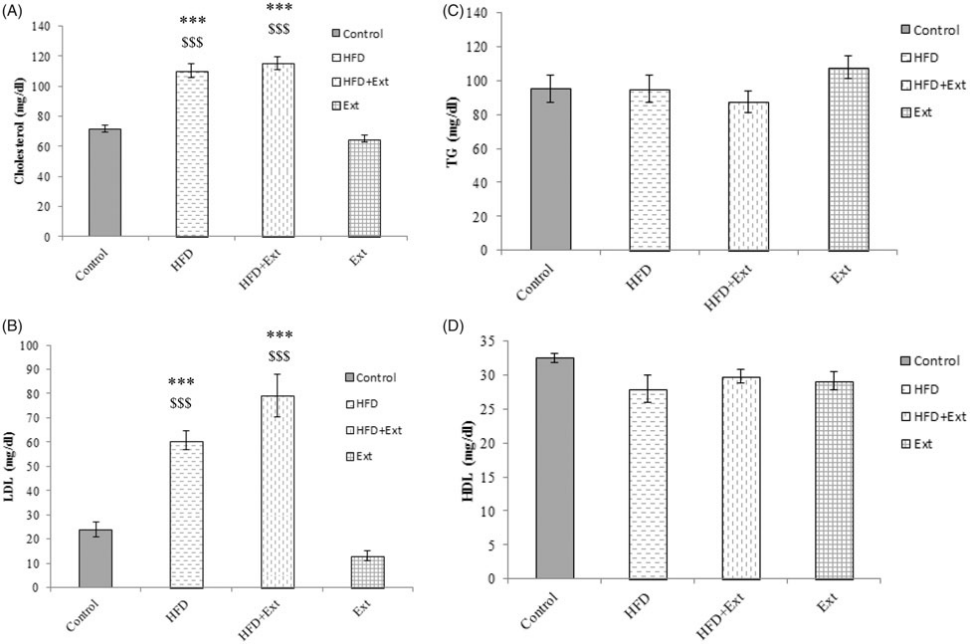
(A) Serum cholesterol, (B) low-density lipoprotein (LDL), (C) triglyceride (TG) and (D) high-density lipoprotein (HDL) levels in the control, high-fat diet (HFD), extract (Ext) and HFD + Ext groups. The HFD and HFD + Ext groups showed higher cholesterol and LDL levels than the control group. ***Statistically significant differences in the HFD and HFD + Ext groups compared to the control group (*p* < 0.001). $$$Statistically significant differences between the HFD, HFD + Ext and extract groups (*p* < 0.001).

After bloodletting from the orbital sinus, the lipid profile was measured in the laboratory. A one-way ANOVA showed that there are significant differences in serum cholesterol between the groups. A Tukey test indicated that there were significant differences in both HFD (110.63 ± 4.59) and HFD + Ext (115.4 ± 4.16) groups compared to the control (71.25 ± 2.83) group (*p* < 0.001). In addition, there were significant differences in both HFD (110.63 ± 4.59) and HFD + Ext (115.4 ± 4.16) groups compared to the extract (65 ± 2.3) group (*p* < 0.001) (Figure 2(A)).

A one-way ANOVA showed that there are significant differences in serum LDL between groups. A Tukey test indicated that there are significant differences in both HFD (60.66 ± 3.88) and HFD + Ext (79.26 ± 8.85) groups compared to the control (23.83 ± 3.05) group (*p* < 0.001). In addition, there were significant differences in both HFD (60.66 ± 3.88) and HFD + Ext (79.26 ± 8.85) groups compared to the extract (15.47 ± 2.07) group (*p* < 0.001) (Figure 2(B)). There were no significant differences between the groups in serum TG (Control: 95.38 ± 8.01; HFD: 95.17 ± 7.95; HFD + Ext: 87.67 ± 6.43; Ext: 107.83 ± 6.53) and HDL (Control: 32.56 ± 0.66; HFD: 28 ± 1.96; HFD + Ext: 29.88 ± 1.04; Ext: 29.14 ± 1.37) levels (*p* > 0.05) (Figure 2(C,D)).

### Effects of *R. damascena* extract and HFD on passive avoidance learning

**Figure 3.**
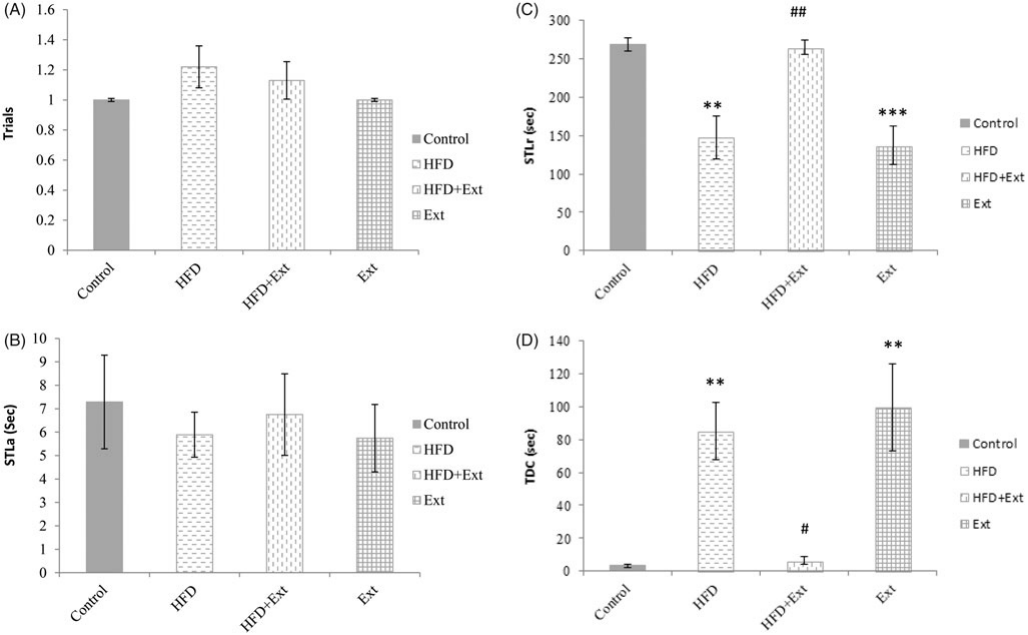
Number of trials to achieve learning in the passive avoidance test (A) step-through latency in the acquisition trial (STLa), (B) step-through latency in the retention trial (STLr) (C) time spent in the dark compartment (TDC) (D) of the passive avoidance learning test. Con = control group, Ext = extract group, HFD = High-fat diet group, HFD + Ext = High-fat diet + extract group. The HFD and extract groups showed statistically significant decreases in step-through latency in the retention test and statistically significant increases in TDC compared to the control group (**p < 0.01 and ***p < 0.001, respectively). The HFD + Ext group showed a statistically significant increase in step-through latency in the retention test compared to the HFD group (#*p* < 0.05; ##*p* < 0.01, respectively).

A one-way ANOVA showed that there are no significant differences in the numbers of trials to acquisition measured among the experimental and control groups (Control: 1 ± 0; HFD: 1.22 ± 0.02; HFD + Ext: 1.13 ± 0.18; Ext: 1 ± 0) of rats (Figure 3(A)). In addition, no significant differences were observed among the different experimental groups in the STLa in the first acquisition trial (before receiving the electrical shock) (Control: 7.29 ± 2.03; HFD: 5.89 ± 1.04; HFD + Ext: 6.75 ± 1.53; Ext: 5.74 ± 1.38) (Figure 3(B)).

The retention test, which was conducted 24 h after training, revealed a significant difference in the STLr among the different groups (Figure 3(C)). Specifically, the STLr of the HFD (150.3 ± 25.2) (*p* < 0.01) and Ext (147.4 ± 23.3) (*p* < 0.001) groups were significantly lower than the respective control (270.4 ± 10.5) groups. STLr was significantly higher in the HFD + Ext group (265.3 ± 10.6) than in the HFD group (150.3 ± 25.2) (*p* < 0.01).

We also observed a statistically significant difference in the TDC between the experimental groups (Figure 3(D)). TDC in the HFD (85.8 ± 19.1) (*p* < 0.01) and Ext-treated (101.4 ± 31.7) (*p* < 0.01) groups was significantly greater than that of the respective control groups (3.1 ± 0.5). TDC in the HFD + Ext group (5.3 ± 2.6) was significantly lower than that in the HFD (85.8 ± 19.1) (*p* < 0.05) and Ext (101.4 ± 31.7) (*p* < 0.05) groups.

### Effects of *R. damascena* extract and HFD on Morris water maze performance

**Figure 4.**
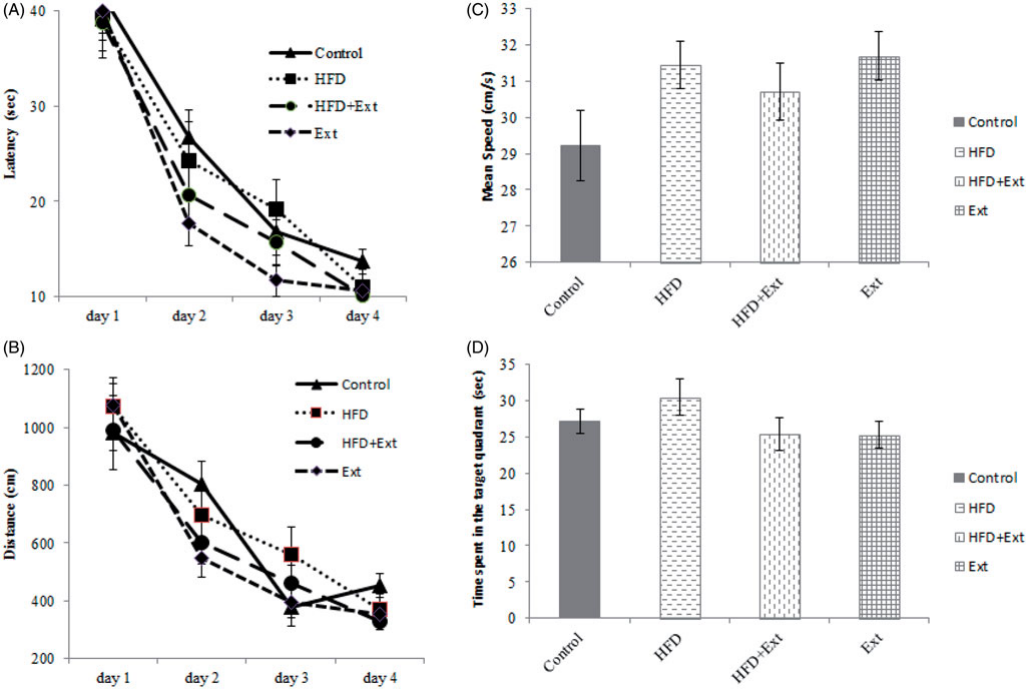
(A) Escape latency, (B) distance travelled, (C) mean swimming speed and (D) time spent in the target quadrant in the Morris water maze test. Con = control group, Ext = extract group, HFD = High-fat diet group, HFD + Ext = High-fat diet + extract group. There were no significant differences between groups.

Our results indicated that there were no significant differences groups between groups in escape latency (Data for day 4: Control: 13.72 ± 1.29; HFD: 11.03 ± 1.89; HFD + Ext: 10.12 ± 1.09; Ext: 10.58 ± 1.09) in the Morris water maze test (Figure 4(A)) (*p* > 0.05). This indicates that rats in all test groups were able to learn the task. Furthermore, there were no significant difference groups between groups in distance travelled (Data for day 4: Control: 452.14 ± 41.1; HFD: 369.91 ± 55.33; HFD + Ext: 329.76 ± 28.66; Ext: 351.66 ± 24.98) and swimming speed (Control: 29 ± 1.6; HFD: 31.51 ± 1.59; HFD + Ext: 30.66 ± 1.57; Ext: 31.77 ± 1.47) (Figure 4(B,C)) (*p* > 0.05). There were no significant differences groups between groups in the time spent in the target quadrant (Control: 27.18 ± 1.69; HFD: 30.49 ± 2.54; HFD + Ext: 25.44 ± 2.29; Ext: 25.32 ± 1.89) during the probe trial (Figure 4(D)) (*p* > 0.05). This indicates that neither HFD nor *R. damascena* affected spatial memory.

## Discussion

This study assessed the influence of the administration of the hydroalcoholic extract of *R. damascena* on learning and memory using the passive avoidance task and Morris water maze performance in HFD-treated rats. HFD decrease STLr and increases TDC in the HFD groups. *R. damascena* administration was able to counteract the negative effects of HFD on memory. The fact that there were no differences in the numbers of trials required for the acquisition of the PAL task indicates that HFD and the hydroalcoholic extract of *R. damascena* have no effects on acquisition. Our results thus indicate that administration of *R. damascena* extract improves HFD-induced memory deficits, as measured by the PAL test. However, *R. damascena* extract had no effects on spatial memory in rats that were fed an HFD for 3 months, as measured by the Morris water maze test. Our finding is supported by several studies that demonstrate the negative effect of HFD on learning and memory. In line with our findings, it has been reported that HFD can impair cognitive function (Komaki et al. 2015; Asadbegi et al. 2017) and synaptic plasticity (Karimi et al. 2015; Asadbegi et al. 2016). Moreover, a recent investigation supported the link between high fat intake and cognitive impairment by revealing that HFD reduces hippocampal BDNF, neuronal plasticity and learning in rats (Molteni et al. 2002; Zhang et al. 2005). Accordingly, saturated fat, hydrogenated fat, and cholesterol can profoundly impair memory and hippocampal morphology (Granholm et al. 2008).

In this study, HFD increased cholesterol and LDL levels in HFD-fed animals. Increases in cholesterol and LDL levels have been reported following the suppression of receptor-dependent LDL transport in the liver (Spady and Dietschy 1988). Moreover, HFD has been shown to be associated with increased serum and liver cholesterol levels and to promote cholesterol accumulation and lipid peroxidation changes in the hippocampus (Stranahan et al. 2011). The absence of an effect of HFD on HDL and TG levels was likely due to the type of diet and the duration of its administration. A similar absence of an effect was observed in previous studies, where methanol extracts of *R. damascena* had no effect on TG, cholesterol, LDL or HDL levels in rabbits consuming an HFD for 45 d (Gholamhoseinian et al. 2012; Joukar et al. 2013). The absence of an effect of the ethanol extract of *R. damascena* on cholesterol and LDL levels in the HFD + extract group in our study may result from variations in saturated fatty acids (e.g., ghee and hydrogenated oil) and high levels of cholesterol and carbohydrates in the HFD that was used.

In our study, the positive effects of *R. damascena* extract on passive avoidance learning in the HFD + extract group were likely due to its antioxidant properties. Studies have shown that HFD causes oxidative stress in the brain and enhances the formation of reactive oxygen species (ROS) (Zhang et al. 2005; Kumar et al. 2014). Many experiments have advised the utilization of antioxidant supplementation to decrease oxidative stress levels and slow down or prevent the improvement of disease-associated complications (Tandon and Gupta 2005; Kizil et al. 2008). In our experiment, total phenolic and flavonoid contents of *R. damascena* were 3110.10 ± 10.20 and 1240 ± 14.5 mg per 100 g of extract, respectively. Flavonoids are described to act as antioxidants (Pignol et al. 1988; Cook and Samman 1996). Their high antioxidant capacity is attributed to their potential of scavenging ROS, chelating metal ions, inhibiting lipid peroxidation and other free radicals, which are produced from different cellular reactions and lead to oxidative stress (Bors et al. 1990). Oxidative stress has been shown to play an important role in cognitive impairment (Serrano and Klann 2004; Alzoubi et al. 2013). Some studies have noted that oxidative stress induces neural damage in brain areas implicated in the aetiology of memory impairment (Shoham et al. 2007; Caceres et al. 2010; Dumont and Beal 2011). According to a previous study, the major components of *R. damascena* are citronellol, geraniol, linalool, kaempferol and quercetin (Kalim et al. 2010). These substances have antioxidant properties (Brewer 2011; Slima et al. 2013; Farhath et al. 2015). Another study found that *R. damascena* provides protection against oxidative DNA damage through its potentially significant antioxidant effects. This suggests that *R. damascena* may be exploited as a potential source of natural antioxidant compounds (Achuthan et al. 2003; Kalim et al. 2010). Other studies have shown that *R. damascena* is a potent antioxidant with many therapeutic uses and that supplementation with *R. damascena* extract results in significant decreases in mortality rates among male and female *Drosophila melanogaster* by decreasing oxidative stress (Jafari et al. 2008; Kalim et al. 2010). Our results, which indicate the presence of memory deficits in rats receiving *R. damascena* extract is unexpected and likely results from the extensive elimination of ROS, which contributes to molecular signalling (Hancock et al. 2001). In a previous study, tempol administration impaired learning and memory in healthy rats but improved the performance of diabetic rats on the PAL test (Jabbarpour et al. 2014). These contradictory effects of tempol were related to its strong antioxidant effects (Wilcox 2010) and the extensive elimination of ROS (Jabbarpour et al. 2014).

HFD has been shown to impair hippocampal neurogenesis through the elevation of lipid peroxidation (Park et al. 2010). On the other hand, quercetin, a flavonoid found in *R. damascena*, stimulates the formation of new dendrites and increases hippocampal volume, density and neurogenesis, which may be beneficial in the elderly (Esfandiary et al. 2014).

A previous study demonstrated that compounds in *R. damascena,* such as geraniol, citronellol, nerol and particularly phenyl ethyl alcohol, inhibit acetylcholinesterase and thereby increase acetylcholine levels (Senol et al. 2013). According to several studies, the effects of acetylcholine on the brain’s cholinergic system increase the perceived positive effect on memory (Buccafusco et al. 2005; Eidi et al. 2006).

In the current study, *R. damascena* extract exerted positive effects on PAL but had no effects on spatial memory. These results are likely due to differences in cell signalling mechanisms and the neuronal circuits involved in each task. Spatial memory depends on perilimbic-hippocampal circuits, while passive avoidance learning is mediated by hippocampal networks other than the perilimbic-hippocampus circuit (Wang and Cai 2008).

## Conclusions

In conclusion, the present study clearly demonstrates that treatment with the ethanol extract of *R. damascena* can prevent cognitive impairment caused by the consumption of an HFD, as measured by the passive avoidance learning test. These effects are likely due to the strong antioxidant properties of the extract and its ability to scavenge free radicals. However, *R. damascena* extract had no effect on spatial memory. This result may be due to the differences in cell signalling properties and the neuronal circuits involved in passive avoidance learning and spatial memory.
